# Looking at the evidence in visual world: eye-movements reveal how bilingual and monolingual Turkish speakers process grammatical evidentiality

**DOI:** 10.3389/fpsyg.2015.01387

**Published:** 2015-09-15

**Authors:** Seçkin Arslan, Roelien Bastiaanse, Claudia Felser

**Affiliations:** ^1^International Doctorate for Experimental Approaches to Language and Brain, University of GroningenGroningen, Netherlands; ^2^Research Group Neurolinguistics, Center for Language and Cognition Groningen (CLCG), University of GroningenGroningen, Netherlands; ^3^Potsdam Research Institute for Multilingualism, University of PotsdamPotsdam, Germany

**Keywords:** evidentiality, information source, inference, witnessing, visual world paradigm, eye-movements, Turkish-German bilingualism

## Abstract

This study presents pioneering data on how adult early bilinguals (heritage speakers) and late bilingual speakers of Turkish and German process grammatical evidentiality in a visual world setting in comparison to monolingual speakers of Turkish. Turkish marks evidentiality, the linguistic reference to information source, through inflectional affixes signaling either direct (-DI) or indirect (-mIş) evidentiality. We conducted an eye-tracking-during-listening experiment where participants were given access to visual ‘evidence’ supporting the use of either a direct or indirect evidential form. The behavioral results indicate that the monolingual Turkish speakers comprehended direct and indirect evidential scenarios equally well. In contrast, both late and early bilinguals were less accurate and slower to respond to direct than to indirect evidentials. The behavioral results were also reflected in the proportions of looks data. That is, both late and early bilinguals fixated less frequently on the target picture in the direct than in the indirect evidential condition while the monolinguals showed no difference between these conditions. Taken together, our results indicate reduced sensitivity to the semantic and pragmatic function of direct evidential forms in both late and early bilingual speakers, suggesting a simplification of the Turkish evidentiality system in Turkish heritage grammars. We discuss our findings with regard to theories of incomplete acquisition and first language attrition.

## Introduction

Evidentiality refers to the linguistic encoding of the type of information source an event description is based on, such as whether or not the event has been witnessed directly by the speaker (Aikhenvald, 2004). Most languages express evidentiality through lexical adverbs (e.g., *reportedly*). However, in Turkish, evidentiality is conveyed through verb inflections requiring the speaker to distinguish whether an event has been directly witnessed or has been indirectly inferred or reported (Slobin and Aksu, 1982). In this study, we provide pioneering data on how grammatical evidentiality is processed by adult Turkish monolinguals, early bilinguals (i.e., heritage speakers of Turkish), and late bilinguals (i.e., L2 learners of German) in an eye-tracking-during-listening experiment.

Effects of bilingualism on one’s native language are subject to a number of variables; in the current study, we will focus on the onset of bilingualism. Two types of bilinguals are of interest in this respect: early bilinguals (heritage speakers of a minority language) and bilingual individuals who learnt the dominant majority language after childhood. A possible consequence of bilingualism is the selective loss of properties of an individual’s first language. Verbal morphology and certain syntactic constraints have been shown to be susceptible to selective erosion (‘attrition’) after full acquisition of the first language (De Bot and Weltens, 1991; Seliger and Vago, 1991; Yağmur, 1997; Cook, 2003; Gürel, 2004; Köpke and Schmid, 2004; Pavlenko, 2004; Köpke et al., 2007; Sorace and Serratrice, 2009). First language attrition has specifically been associated with late bilingualism. In early bilinguals (in particular, ‘heritage speakers’), properties of the first language have instead been argued to be prone to disrupted acquisition processes during childhood (e.g., Montrul, 2002, 2008, 2009; Polinsky, 2006; Albirini et al., 2011, 2013). That is, early bilinguals are often assumed to not have reached full acquisition of several properties of the heritage language, due to reduced input conditions.

Köpke (2004) defines attrition as the “loss of the structural aspects of the language, i.e., change or reduction in form”. In bilingual acquisition contexts, first language attrition is a possible outcome in bilinguals who acquired their second language later in life (e.g., after puberty), and after fully acquiring their first language during childhood (De Bot and Weltens, 1991; Seliger and Vago, 1991; Yağmur, 1997; Cook, 2003; Gürel, 2004; Köpke, 2004; Pavlenko, 2004; Tsimpli et al., 2004; Köpke et al., 2007). In contrast to language attrition in late bilinguals, Montrul (2002, 2008) and Polinsky (2006) have shown that an early onset of bilingualism may lead to incomplete acquisition, that is, to a failure in acquiring part(s) of the first language grammar during early childhood. Incomplete acquisition has mainly been observed in heritage speakers, who during childhood were exposed to their first language within a minority population away from where that language is spoken natively. Studies on heritage speakers of Spanish (Montrul, 2002, 2008, 2009), Russian (Polinsky, 2006, 2008), and Arabic (Albirini et al., 2011, 2013) have confirmed that several aspects of the first language grammar are subject to divergent performance and/or competence from monolingual speakers.

Montrul (2002, 2008) suggests that a disrupted acquisition process may result in unsuccessful ultimate attainment of the inherited (first) language in early bilingual adults, and that the effects of incomplete acquisition may be more severe compared to the effects of first language attrition in late bilinguals. Incomplete acquisition does not seem to affect all areas of inflectional morphology equally, however. Montrul (2009), for example, investigated adult Spanish heritage speakers’ sensitivity to aspectual (preterit – imperfect) and modal (subjunctive – indicative) distinctions using an elicited oral production task, a written morphology recognition task, and a judgment task. She found that the heritage speakers’ knowledge of aspectual distinctions was better retained than their knowledge of modal distinctions, suggesting that the heritage speakers were affected by incomplete acquisition of Mood. Given that Aspect tends to be acquired earlier than Mood, Montrul (2009) attributes the heritage speakers’ greater problems with Mood to maturational factors (i.e., the order of acquisition of inflectional distinctions).

Montrul’s (2009) observation of Mood distinctions being eroded more than aspectual ones in Spanish heritage language is consistent with Jakobson’s (1941) Regression Hypothesis, which holds that linguistic properties that are acquired late will be lost first (see Keijzer, 2010). Montrul’s findings are also compatible with the Interface Hypothesis (Sorace, 2000; Sorace and Filiaci, 2006; Sorace and Serratrice, 2009), according to which linguistic properties at ‘interfaces’ (e.g., syntax–discourse interface) may prove particularly problematic in bilingual acquisition. Linking syntactic and discourse-level information is claimed to be particularly difficult. Sorace and Serratrice (2009) argue that “bilinguals may have fewer processing resources available and may therefore be less efficient at integrating multiple types of information in on-line comprehension and production at the syntax – pragmatics interface.” Therefore, even highly proficient bilinguals may show difficulty using or processing grammatical forms that are marked in the sense of requiring very specific pragmatic licensing conditions. Sorace (2011), however, cautions against extending the Interface Hypothesis, which originally sought to account for non-target like performance patterns in near-native second language speakers, to heritage speakers.

In past few years, there has been increasing interest in understanding the properties of subtractive bilingualism, when the first language is a minority language. Most previous studies have focused on early and late bilinguals (i.e., heritage speakers and L2 speakers) living in the U. S. The nature of language erosion in bilingual individuals living in Western Europe is less well understood. Turkish is one of the most widely spoken minority languages in Germany, and it differs typologically from most of the previously studied heritage languages. Turkish is an agglutinative language with rich inflectional morphology, including the grammatical expression of evidential distinctions. The linguistic features of Turkish evidentials are described in more detail below, as well as previous experimental studies on this phenomenon.

### Evidentiality in Turkish

Evidentiality refers to the linguistic encoding of a particular type of evidence for a speaker’s utterance (Chafe and Nichols, 1986; Willett, 1988; Lazard, 2001; Plungian, 2001; Aikhenvald, 2004). The nature of the evidence relates to how a speaker has access to the information in his or her statement: witnessing, inference, or hearsay. Turkish expresses evidentiality through a verbal inflection paradigm with two choices for direct (witnessing) and indirect evidence (inference or hearsay), as illustrated in (1) and (2), respectively.

(1) Adam elmayı ye**di**
    man apple_ACC_ eat _DIRECT EVIDENTIAL_
    ‘The man ate the apple’ [witnessed]
(2) Adam elmayı ye**miş**
    man apple_ACC_ eat _INDIRECT EVIDENTIAL_
    ‘The man ate the apple’ [reported or inferred]

The direct evidential suffix –DI is used to refer to past events that were directly witnessed, or participated in, by the speaker. For example, in (1) –DI signals that the speaker has witnessed the apple being eaten. The indirect evidential suffixes –mIş and –(I)mIş are appropriate for use in inference or reportative contexts, respectively. For instance, in (2) the speaker has been either told that the man ate the apple, or has (physical) evidence leading him or her to infer that the man ate the apple, such as seeing peelings and leftovers of an apple on the table.

In inference contexts, the use of an indirect evidential signals non-witnessed past events that are perceived through present states or results on the basis of physical or visual evidence (Aksu-Koç and Slobin, 1986). In reportative contexts it conveys that the information is known through ‘hearsay’ or verbal report from a third party (Slobin and Aksu, 1982). These semantic and formal distinctions in Turkish evidentials are well understood. Several studies have indicated that the indirect evidential is the marked term on the basis of its semantic complexity since it refers to different information sources (i.e., inference and report), whilst the direct evidential is the unmarked form for referring to witnessed past events (Slobin and Aksu, 1982; Aksu-Koç and Slobin, 1986; Aksu-Koç, 1988, 2000; Sezer, 2001; Johanson, 2006). These authors also agree that while the indirect evidential bears epistemically modal connotations, the direct evidential is a non-modal term.

The use of evidentials in interrogative contexts has not been explored much in Turkish linguistics. Aikhenvald (2004) claims that evidentials in an interrogative clause reflect the type of information source available to the questioner or to the addressee. This indicates that the semantic and pragmatic uses of evidentials differ in declarative and interrogative contexts. In *wh-*interrogative clauses such as (3) and (4) below, for example, the use of a particular evidential reflects the type of information source available to the addressee of the question, while the questioner may not necessarily have access to the same information source.

(3) Hangi adam elmayı ye**di**?
    which man apple_ACC_ eat_DIRECT EVID_
    ‘Which man ate the apple?’
(4) Hangi adam elmayı ye**miş**?
    which man apple_ACC_ eat_INDIRECT EVID_
    ‘Which man ate the apple?’

The questioner’s choice of a particular evidential form indicates that he or she is making assumptions on the information source available to the addressee. In (3), the questioner assumes that the addressee has witnessed who has eaten the apple; thus, a direct evidential is used. In (4), by contrast, the questioner presumes that the addressee has access to information about the event through an indirect source (e.g., inference or hearsay), hence, an indirect evidential is used. Therefore, a particular evidential is selected in an interrogative clause depending on what the questioner assumes as to how the addressee may have acquired knowledge of the event concerned.

### Experimental Studies on Turkish Evidentials

Experimental studies on evidentiality in mono- and bilingual Turkish speakers are scarce. The psycholinguistic understanding of grammatical evidentiality is limited to developmental studies in monolingual children and a small number of studies on adult bilinguals. One of the earliest empirical studies was conducted by Aksu-Koç (1988), who examined the production and comprehension of evidential morphology (among other morphemes) in Turkish-speaking children (aged 3–6). She found that the direct evidential morpheme was one of the first to be acquired, followed by the indirect evidential morpheme after a delay of about few months. Aksu-Koç (1988) notes, however, that children’s early use of evidential morphemes tends to be limited to directly perceived events or present states, and that at this developmental stage children may not yet be able to distinguish the direct vs. indirect information contrast. This was confirmed by more recent studies. Öztürk and Papafragou (2007), for example, studied young monolingual Turkish children (aged 3–6) using elicited production and semantic and pragmatic comprehension tasks. The children used evidential forms appropriately but tended to have difficulty distinguishing the semantic and pragmatic content signaled by these forms. In a later study, Öztürk and Papafragou (2008) examined Turkish children (aged 5–7) using both an elicited production and a non-linguistic source monitoring task. The data reveal that Turkish children in all age groups are able to produce direct evidential forms almost faultlessly while their use of indirect evidential develops with age. Inferred and reported information sources proved more difficult for children than directly witnessed information sources even in the oldest age group; see also Ünal and Papafragou (2013). Aksu-Koç (1988) reports that monolingual Turkish children tend to gain control over the semantic and pragmatic content of direct evidentials around the age of three. The inferential readings related to the indirect evidential, however, only stabilize around the age of four in monolingual children, while reportative contexts develop around the age of four and a half. Aksu-Koç et al. (2014) and Aksu-Koç (2014) argue that modal distinctions (including epistemic readings associated with indirect evidentials) are acquired later, and that children at earlier stages of development produce non-modalized markers instead, such as the direct evidential.

Some recent studies show that evidentiality is susceptible to erosion or incomplete acquisition in Turkish heritage speakers. Arslan et al. (submitted) studied Turkish/Dutch early bilingual (i.e., second-generation heritage speakers) and Turkish monolingual adults using a sentence-verification task where participants listened to sentences containing evidential verb forms that mismatched the information contexts. For instance, an indirect evidential was mismatched to ‘seen’ information contexts (*Yerken gördüm, az önce adam yemeǧi yemiş* ‘I saw the man eating; he ate_INDIRECT EVIDENTIAL_ the food’) and a direct evidential was mismatched to ‘heard/indirect’ information contexts (*Yerken görmüsler, az önce adam yemeği yedi* ‘They saw the man eating; he ate_DIRECT EVIDENTIAL_ the food’). Participants’ sensitivity to evidential verb forms was measured by asking them to press a button when a sentence was incongruent. Arslan et al. (submitted) demonstrated that the bilinguals were largely insensitive to both types of evidential mismatches. Interestingly, however, the bilinguals retained their sensitivity to tense violations (i.e., violations by past and future participles without evidentiality marked). Arslan et al.’s (submitted) data showed that evidentiality is a particularly vulnerable part of Turkish grammar in early bilingual speakers.

Furthermore, Arslan and Bastiaanse (2014) investigated narrative speech production in second-generation Turkish/Dutch early bilingual adults. The early bilinguals made a large number of substitution errors by inappropriately using direct evidentials in contexts that required an indirect evidential form. The early bilingual adults showed reduced sensitivity to the semantic distinctions between information sources that the evidential forms signal. Arslan and Bastiaanse (2014), nonetheless, report that the early bilingual adults did not substitute the indirect evidential where a direct one should be produced. The authors suggest that the indirect information source is incorporated while direct evidence is ignored, as if the direct evidential does not carry an evidential value in early bilingual Turkish speakers’ oral production.

Summarizing, previous studies indicate (i) that the direct evidential is acquired earlier than the indirect evidential, possibly due to the latter being more complex in terms of its semantics (e.g., Aksu-Koç, 1988; Öztürk and Papafragou, 2007, 2008); (ii) that evidential terms in Turkish are highly susceptible to erosion in adult heritage speakers (Arslan and Bastiaanse, 2014; Arslan et al., submitted). The studies discussed above have also left some questions unexplored. First, it is not clear whether insensitivity to evidentiality distinctions is restricted to early bilingual heritage speakers or whether it can also be observed in late bilinguals. Second, although Arslan et al. (submitted) measured the processing of evidentiality using a response-time task, the moment-by-moment time course of processing evidentiality has not been investigated yet. Finally, recall that the use of evidential forms is linked to the kind of evidence available to the speaker (in declarative clauses) or the addressee (in interrogative clauses), and nothing is known as yet about how comprehenders interact with this evidence during their processing of grammatical evidentiality.

In the current study, we carried out an eye-movement monitoring experiment with three groups of participants: early and late Turkish/German bilinguals and a reference group of monolingual Turkish speakers. Testing two different bilingual groups should allow us to explore whether differences in the age of bilingualism onset affects bilinguals’ processing of evidentiality. The aim of the experiment was to unveil the nature of processing evidentiality through monitoring participants’ eye movements while they listened to sentences with grammatical evidentiality in a visual-world paradigm. This is a very compelling way to test processing of evidentiality as the visual-world paradigm allows us to measure participants’ moment-by-moment eye-movements while they interact with different types of visual evidence. Our visual stimuli included picture pairs that encoded either ‘witnessed’ or ‘inferable non-witnessed’ events, which were appropriate for the use of direct and indirect evidential forms, respectively. In particular, we sought to answer the following questions:

• Do early and late bilinguals differ from monolinguals in their processing of evidentiality?
• Do monolingual, early and/or late bilingual Turkish speakers differ in their processing of direct and indirect evidentials?

Given the findings of previous studies on early bilingual heritage speakers living in the U. S., inflectional morphology seems to be particularly affected. This is consistent with Arslan et al.’s (submitted) findings for early bilingual speakers of Turkish in the Netherlands. Considering these data, we expect early bilinguals to show a reduced sensitivity to evidentiality in comparison to monolingual Turkish speakers. If this is a consequence of incomplete acquisition, then early bilinguals will also be sensitive to evidentiality compared to late bilinguals, who we expect to pair with the monolinguals. The hypotheses we introduced above moreover predict an asymmetrical insensitivity in bilingual participants’ responses to direct and indirect evidential forms. Specifically, the Interface Hypothesis predicts more problems during bilinguals’ processing of the indirect than the direct evidential forms. According to this hypothesis, integrating information from multiple linguistic domains – in particular, integrating morphosyntactic and pragmatic information – is difficult for speakers who have not fully acquired the language under investigation. Recall that the use of indirect evidentials is licensed only in specific pragmatic contexts that require more or less complex inferential reasoning, whereas direct evidentials are used as an ‘elsewhere’ form in the absence of such contexts, signaling that an event was witnessed directly. The Regression Hypothesis also predicts more problems in bilinguals’ responses to indirect than to direct evidential forms as the former are acquired later in development.

## Materials and Methods

### Participants

Sixty-one adult Turkish speakers were recruited from the Turkish community of Berlin, Germany. They were categorized into three groups on the basis of their age of onset of bilingualism. A group of early bilinguals (*n* = 19), who were all born in Germany (i.e., second generation heritage speakers of Turkish), and a group of late bilinguals (*n* = 20) were recruited. The late bilinguals were L2 learners of German who came to Berlin after puberty (i.e., after the age of 13). Finally, a reference group of monolingual Turkish speakers (*n* = 22) who had no previous contact with German also participated. A demographic information questionnaire was completed by all participants. In addition, the bilinguals responded to a short language test in both German and Turkish, adapted from the Goethe (Goethe-Institut e.V.) and telc (telc GmbH) placement tests; see **Table 1**.

**Table 1:** Numbers and age of participants, AoA = age of acquisition in years with min-max age range, and proficiency test scores (ranges in brackets) in Turkish and German for bilingual participants.

	*N*	Age	AoA Turkish	AoA German	Turkish score	German score
Monolingual	22	24 (20–36)	From birth	NA	NA	NA
Late bilingual	20	30 (21–46)	From birth	13–27	89.5% (63–100)	61.3% (23–93)
Early bilingual	19	27 (22–36)	From birth	1–4	71.1% (13–100)	91.2% (76–100)

The monolinguals were native Turkish speakers from Turkey who were in Berlin for holidays or family visits during the time they were recruited. None of them spoke any German. All participants were highly educated (i.e., college students or graduates) and spoke the standard Turkish dialect. No speakers of any ethnical languages or dialects participated in this study. The participants had normal hearing and (corrected to normal) vision. They gave their consent under the Helsinki declaration and were paid a fee of 10 Euros.

### Materials

**FIGURE 1:**
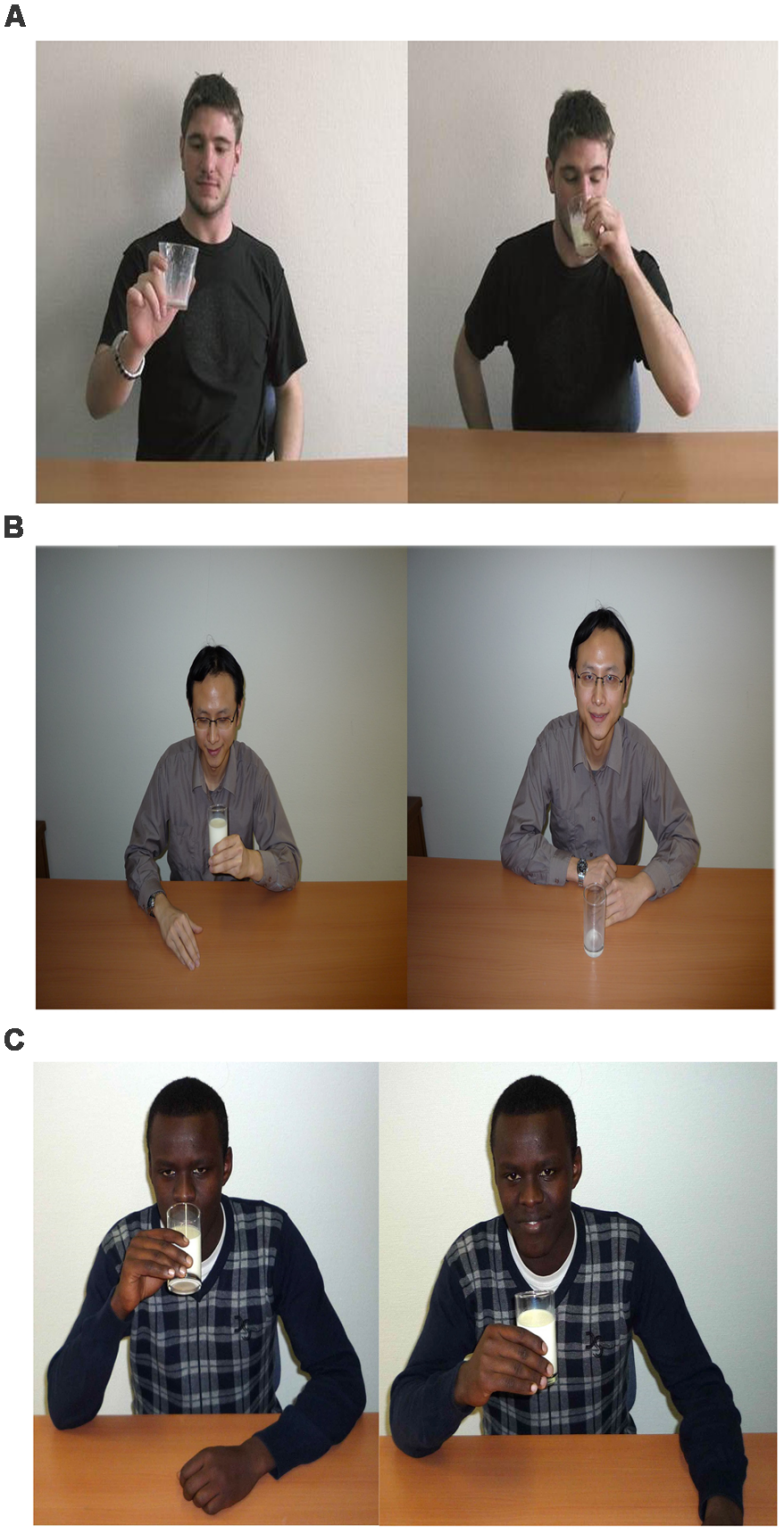
**Examples of visual displays appeared in three different conditions: **(A)** – direct evidential, **(B)** – indirect evidential, **(C)** – future tense. ©Roelien Bastiaanse, University Groningen**.

Sixty visual displays, each comprising a pair of photos presented next to each other, were created as shown in **Figure 1**. One of the photos was the target picture and the other one served as a context picture. To create the visual displays, 20 action verbs were combined with six different people and 10 different inanimate objects (i.e., *süt içmek* ‘to drink milk’). The same actions were displayed in two experimental conditions, a direct and an indirect evidential one, as well as in a non-evidential distractor condition involving the future tense (*n* = 20 each). The photographs used in this experiment were taken from European, Asian, and African versions of the Test for Assessing Reference of Time: TART (Bastiaanse et al., 2008). Different ‘models’ from different versions of TART were used with the same action displayed in different conditions in a counterbalanced manner. For example, drinking milk appeared once in the direct evidential condition acted by a European-looking person, once in the indirect evidential condition acted by a person of Asian appearance, and once in the future tense condition acted by a person of African appearance as shown in **Figure 1**. An equal number of male and female ‘models’ appeared in each condition.

To encode direct and indirect evidentiality contexts visually, different states of the same action were represented next to each other. For the direct evidential condition, an action was shown while it was happening in one of the photographs and its end-state in the other (see **Figure 1A**). This was an example of a witnessed event, appropriate for the use of a direct evidential form. For the indirect evidential condition (**Figure 1B**), an action was displayed in its end-state and in a ‘pre-action’ state, that is, before the action was initiated. This means that the action could only possibly be inferred, making this kind of visual display appropriate for the use of an indirect evidential form. In both evidential conditions, the target picture was the photograph that depicted the end-state of the action. For the future tense condition (**Figure 1C**), an action was shown in the target photo in its pre-action state. The future items also included a context photo, which was showing the action as ongoing in half of the future items, and in its end-state in the other half. The order of the two photographs was reversed in half of the items so that the target picture did not always appear on the same side.

The auditory stimuli consisted of interrogative clauses that were read by a female Turkish native speaker and digitally recorded. Examples for each of the three conditions are given in (5)–(7) below. In the two evidential conditions, the participants were asked to identify the picture showing the result of the action. In the future tense condition, the target picture was the one depicting a pre-action state (e.g., with the glass of milk still full and untouched).

(5) Direct evidential
    Hangi fotoğraftaki adam dün sütü
    which photograph_LOC_ man yesterday milk_ACC_
    iç**ti** ender bir istekle?
    drink_DIRECT EVID_ unusual one desire
    ‘In which photograph did the man drink the milk yesterday with an unusual desire?’
(6) Indirect evidential
    Hangi fotoğraftaki adam dün sütü
    which photograph_LOC_ man yesterday milk_ACC_
    iç**miş** ender bir istekle?
    drink_INDIRECT EVID_ unusual one desire
    ‘In which photograph did the man drink the milk yesterday with an unusual desire?’
(7) Future tense (non-evidential)
    Hangi fotoğraftaki adam birazdan sütü
    which photograph_LOC_ man soon milk_ACC_
    iç**ecek** ender bir istekle?
    drink_FUTURE_ unusual one desire
    ‘In which photograph will the man drink the milk soon with an unusual desire?’

A three-word padding phrase (e.g., *ender bir istekele* ‘with unusual desire’) was added at the end of each interrogative clause to preclude the auditory stimuli from terminating at the critical verb. Extending the stimuli sentences in this way was necessary so as to extend measuring time and thus enable us to capture potential spillover effects, and to reduce the possibility of our eye-movement data being affected by global end-of-sentence wrap-up processes.

### Evaluation of the Experimental Sentence Stimuli^1^

The plausibility of our experimental stimuli was evaluated in an oﬄine rating study using a four-point Likert scale (1 = very plausible, 4 = very implausible). To construct plausible test items, the evidentiality sentences exemplified by (5) and (6) were converted into declarative clauses. The ‘plausible direct evidential condition’ (*n* = 20) contained semantically coherent sentences with a direct evidential form (e.g., *adam dün sütü içti, ender bir istekle* ‘the man drank the milk with unusual desire’), and the ‘plausible indirect evidential condition’ (*n* = 20) contained semantically coherent sentences with an indirect evidential form (e.g., *adam dün sütü içmiş, ender bir istekle* ‘the man drank the milk with unusual desire’). To create implausible counterparts of the plausible conditions, the agent and theme arguments in those sentences were reversed (e.g., *süt dün adamı içti, ender bir istekle* ‘the milk drank the man with unusual desire’). The plausible and implausible sentences were distributed across four presentation lists, counterbalanced across participants. Sentences constructed with a same verb in different conditions appeared in different lists so as to minimize potential effects of repetition. In addition, 30 plausible and implausible filler sentences were added to each list, resulting in a total of 50 items per list.

Participants included 43 monolingual speakers of standard Turkish (mean age = 26.3, range = 17–45, 24 males), none of whom took part in the main eye-tracking experiment. All participants were living in Turkey and none of them reported to speak any foreign language proficiently. The rating task was administered as a web-based questionnaire. At the beginning of the task, the following instructions were provided in Turkish: “You are being asked to rate the plausibility of some Turkish sentences (i.e., how ‘intuitive and reasonable’ do these sentences sound to you). Please read each sentence carefully and click on one of the answer choices provided under each sentence. On every page, there are five sentences. When you have finished rating the sentences on one page, click on ‘continue,’ and when you have finished rating all of the sentences, please click on ‘submit’.”

The results showed that the plausible direct evidential condition was rated significantly more favorably than its implausible counterpart [1.66 vs. 3.73, *t*(42) = -19.4, *p* < 0.0001], and the plausible indirect evidential condition was rated as more plausible than its implausible counterpart [1.60 vs. 3.83, *t*(42) = -23.3, *p* < 0.0001]. Crucially, participants’ ratings of the plausible direct and indirect evidential conditions did not differ statistically [*t*(42) = 1.39, *p* = 0.17], and neither did their ratings of the two implausible conditions [*t*(42) = 1.76, *p* = 0.09].

### Procedure

Presentation of visual and audio stimuli was programmed in two lists by using the SMI experiment builder software (SensoMotoric Instruments GmbH). A participant saw two photos presented next to each other in each trial, as described above. The evidential items were counterbalanced across participants over the two lists, so that an evidential item only appeared in either the direct or the indirect evidential condition. Each participant saw 10 direct and 10 indirect evidential items. In addition, 20 future tense items were added to each list as non-evidential distractor items. Therefore, each participant was exposed to an equal number of evidential and non-evidential items. A further 20 filler items, containing a subject participle complement clause (i.e., a non-finite verb form: *Hangi fotoğraftaki adam dün yemeği pişiren adam* ‘which photograph_LOC_ man yesterday food_ACC_ cook _SUBJECT PARTICIPLE_ man?’), were added so that each presentation list contained 60 items. Presentation of the auditory stimuli was delayed by 1 s with respect to the visual stimuli in all items. Pauses were programmed after every block of 20 items. The items were presented in a randomized manner.

Participants were tested individually in a dedicated testing room in Berlin. They were asked to sit within a convenient sight distance from a 1680 pixels × 1050 pixels-wide (i.e., 22 inches) PC screen. They were then given the following instructions in Turkish: “You are about to begin an eye-tracking experiment. Please listen to the sentences carefully, and click on the photograph that corresponds to the sentences you hear. When you click the next item will begin.” Two practice trials were presented during which the participants were provided with feedback and the opportunity to ask questions if they had any. Before the main eye-tracking experiment began, participants were reminded not to turn their gaze off the screen. When participants responded, the presentation of the next stimulus was initiated manually by the experimenter. Eye movements were monitored and sampled at a rate of 60 Hz, one frame per 16 ms, by a remote SMI eye-tracking system positioned underneath the stimulus screen. The research was approved by the ethics committee of the University of Potsdam (application number 37/2011).

### Analysis

Three types of dependent variables were obtained and analyzed separately: accuracy of clicks, response times (RTs), and proportion of looks. The accuracy data were analyzed using generalized linear mixed-effects regression models, and the RTs data using linear mixed-effects regression models (Baayen, 2008). RTs that exceeded three standard deviations beyond the group means were excluded. Any responses made before the onset of the critical verbs were rejected (around 1.5%). For the proportions of looks analysis, a time window of 2000 ms from the onset of the critical verb was selected.^2^ The first 200 ms after verb onset were excluded from this time window, since it takes about 200 ms to program and execute an eye movement (Rayner et al., 1983). Proportion of looks was a binary variable indicating whether the participants fixated on the target picture or not. We excluded 0.92% of the data due to off-screen looks. The analyses were done on non-aggregated data. Participants’ proportion of looks were analyzed with mixed-effects multilevel logistic regression models (Barr, 2008), using the ‘lme4’ and ‘multcomp’ statistical packages of R version 3.1.1 (R-Core-Team, 2012).

## Results

### Accuracy and Response Times

Mean accuracy and RTs data are shown in **Table 2** and the fixed effects from mixed-effects regression models performed on accuracy and RTs of responses are given in **Table 3**. For the accuracy data, significant effects of group with negative estimate values indicate that both late and early bilinguals were less accurate than monolinguals.^3^^,^^4^ However, the between-groups differences were modulated by condition, as witnessed by significant interactions between the factors group and condition. Therefore, *post hoc* analyses were performed using Tukey tests. These revealed that both late (β = 0.213, *SE* = 0.04003, *z* = 5.326, *p* > 0.001) and early bilinguals (β = 0.228, *SE* = 0.035, *z* = 6.418, *p* < 0.001) responded less accurately to the direct evidential than to the indirect evidential condition, whereas the monolinguals showed no difference between the two conditions (β = 0.0105, *SE* = 0.029, *z* = 0.353, *p* = 0.072). There were group differences in participants’ responses in the direct evidential condition, with both the early (β = -1.897, *SE* = 0.5404, *z* = -3.511, *p* = 0.0012) and the late bilinguals (β = -1.685, *SE* = 0.5311, *z* = -3.172, *p* = 0.0042) less accurate than the monolinguals. The early and late bilinguals did not differ in their responses in the direct evidential condition (β = 0.212, *SE* = 0.5005, *z* = 0.424, *p* = 0.905). For participants’ responses in the indirect evidential condition, no within or between group differences were observed (all *p*s > 0.346).

**Table 2:** Mean proportion of accuracy, standard error rates (*SE*), and response times (RTs) of click responses.

	Monolingual	Late bilingual	Early bilingual
**Accuracy**			
Direct evidential	0.89 (0.02)	0.67 (0.03)	0.63 (0.03)
Indirect evidential	0.89 (0.02)	0.90 (0.02)	0.85 (0.03)
Future tense (distractor)	0.93 (0.01)	0.90 (0.01)	0.88 (0.02)
**RTs**			
Direct evidential	2214.7	2707.4	2716.5
Indirect evidential	2262.3	2339.7	2494.3

**Table 3:** Fixed effects from the generalized linear mixed-effects regression models performed on accuracy of clicks and linear mixed-effects regression model performed on RTs.

	Accuracy of clicks				RTs of clicks			
Fixed effect	Estimate	*SE*	*Z*-value	*p*-value	Estimate	*SE*	*t*-value	*p*-value
Intercept	2.472	0.353	6.994	<0.001***	2282.28	200.04	11.409	<0.001***
Condition (indirect evidential)	0.056	0.310	0.182	0.855	-25.08	107.34	-0.234	0.815
Group (late bilingual)	-1.737	0.407	-4.262	<0.001***	525.21	268.52	1.956	0.050*
Group (early bilingual)	-1.531	0.403	-3.798	<0.001***	554.36	274.24	2.021	0.043*
Condition × Group (late bilingual)	1.393	0.426	3.266	0.001**	-420.22	160.05	-2.626	0.009**
Condition × Group (early bilingual)	1.762	0.443	3.977	<0.001***	-349.25	169.03	-2.066	0.039*
	Formula: accuracy ∼ Condition * Group + (1| subject_no) + (1| item_no)				Formula: RTs ∼ Condition * group + (1| item_no) + (1| subject_no)			

*p < 0.05, **p < 0.01, ***p < 0.001.

With regard to RTs, the model outputs shown in **Table 3** revealed significant effects of group but not of condition. The negative estimate values of the group effects confirm that both late and early bilingual groups were slower in their responses than monolinguals irrespective of condition. Since the interactions between group and condition were also significant, *post hoc* analyses were performed. Both the late (β = 372.10, *SE* = 116.10, *z* = -3.204, *p* = 0.001) and early bilinguals (β = 332.90, *SE* = 150.0, *z* = -2.22, *p* = 0.026) showed longer RTs to the direct evidential condition than to the indirect evidential condition, whereas no significant between-condition difference was seen in the monolinguals (β = -29.31, *SE* = 100.30, *z* = -0.292, *p* = 0.77). Within the responses in the direct evidential condition, group contrasts proved significant. Both the early (β = -475.26, *SE* = 156.45, *z* = -3.038, *p* < 0.01) and the late bilinguals (β = -401.01, *SE* = 150.37, *z* = -2.667, *p* = 0.020) responded slower than the monolinguals, whereas late bilinguals did not differ from the early bilinguals (β = -74.25, *SE* = 168.33, *z* = -0.441, *p* = 0.77). Within the responses in the indirect evidential condition, by contrast, no group differences were found (all *p*s > 0.14).

### Proportions of Looks

**FIGURE 2:**
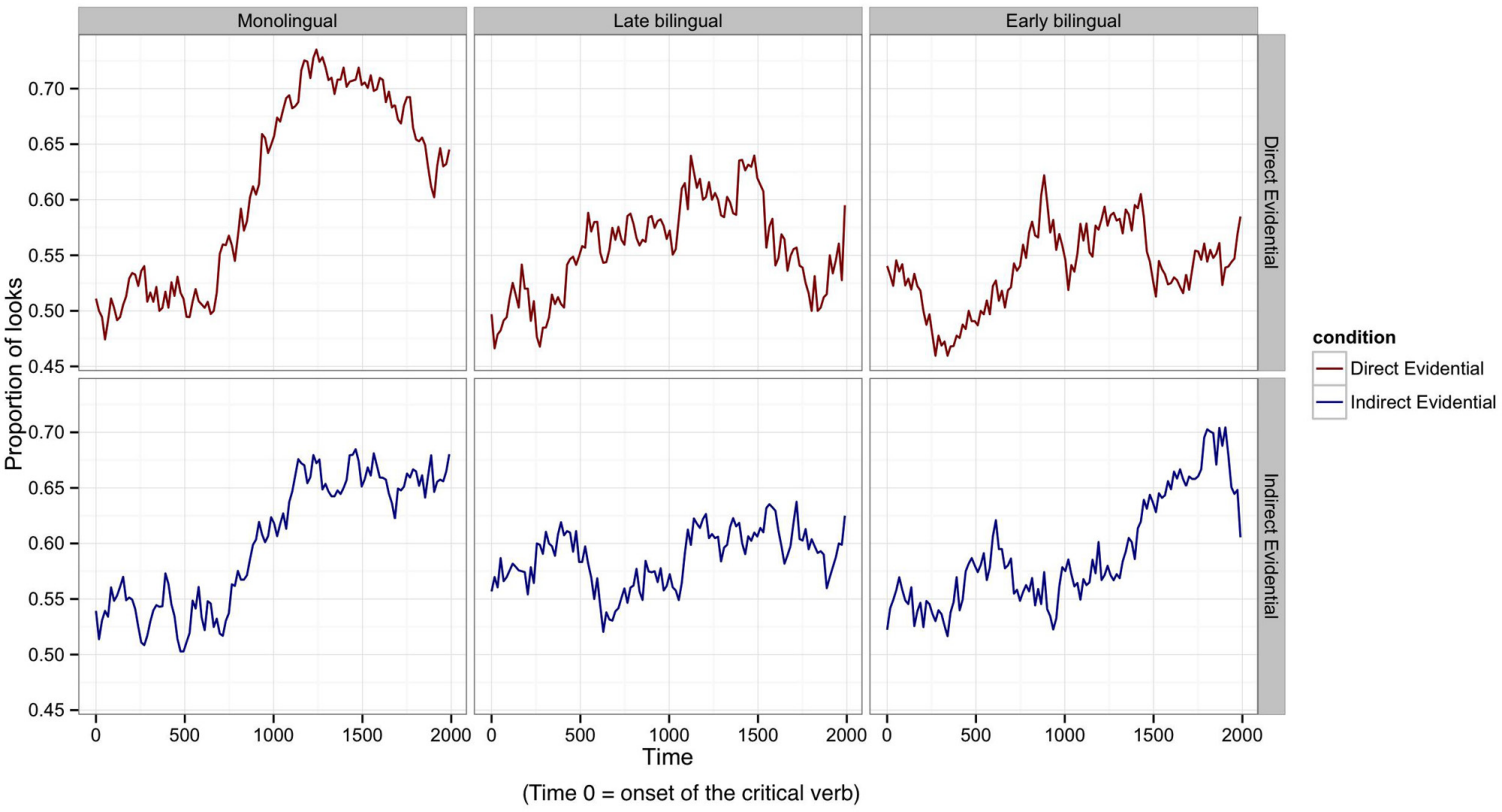
**Mean proportions of target fixations per participant group and condition for the 2000 ms time window from the onset of the critical verb.** The y-axis shows participants’ mean fixation proportions for each of the two evidentiality conditions.

**FIGURE 3:**
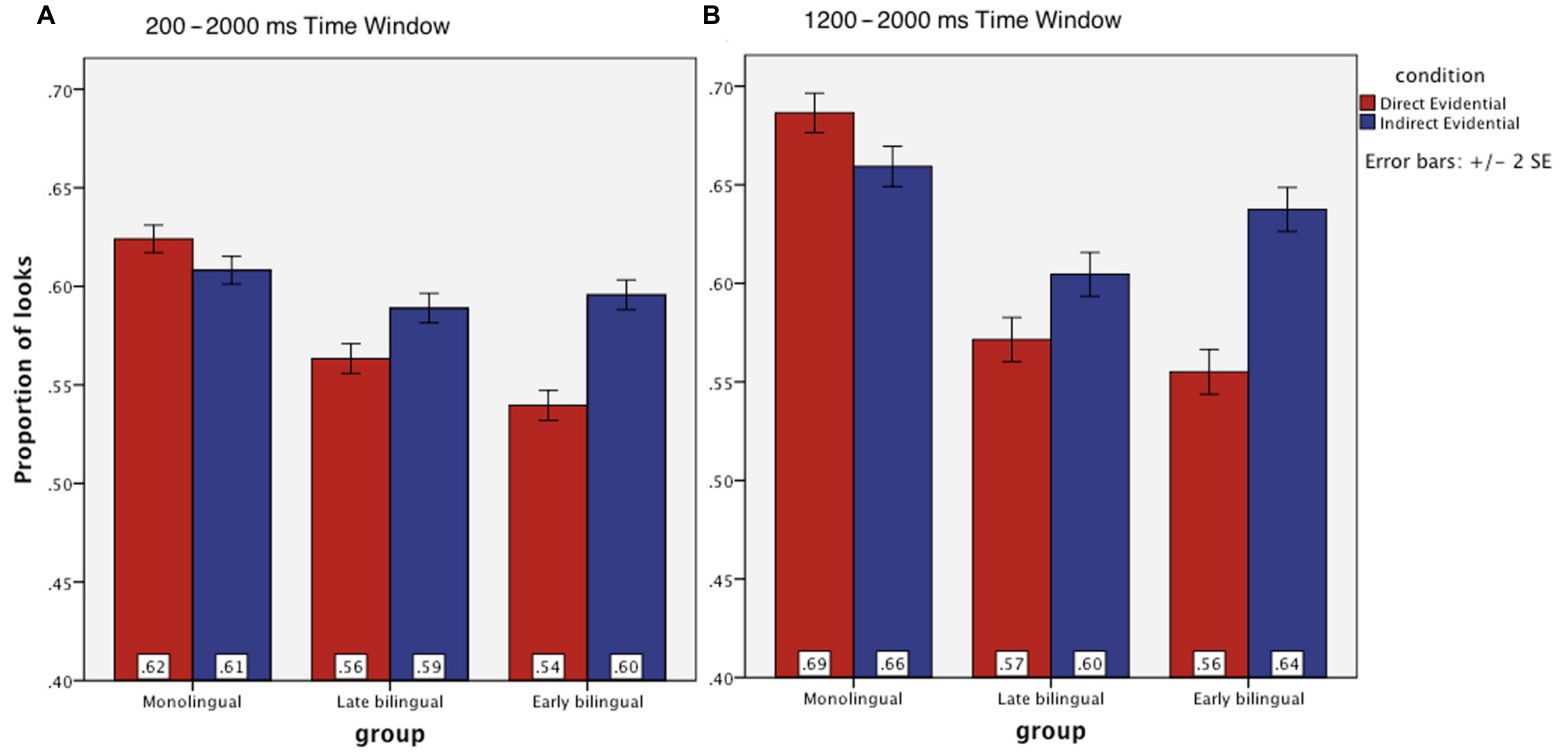
**Mean proportions of target fixations in three groups of participants during their processing of direct and indirect evidentials in two different time windows: **(A)** 200–2000 ms and **(B)** 1200–2000 ms after verb onset**.

**Figure 2** illustrates the moment-by-moment changes in participants’ proportions of looks toward the target picture for the direct and indirect evidential conditions during the entire 2000 ms time window, and **Figure 3** shows the mean proportions of looks in the main and later time windows, respectively. **Figure 2** indicates that the proportions of looks to the target picture were around 50% (i.e., participants gazed on both the target and context photographs with equal likelihood) at the beginning of the time window for all groups, which confirms that participants did not visually prefer one photograph over the other before they heard the critical verb form. As we mentioned above, any fixation changes prior to 200 ms from verb onset cannot be attributed to the critical stimulus.

Visual inspection of the eye-movement data indicated that during the initial 200–1000 ms after verb onset, both bilingual groups’ eye movements tended to oscillate between the target and context pictures, and that a more stable increase in looks to the target picture only emerged after about 1000 ms (see **Figure 2**). The monolinguals, however, showed more stable eye-movement patterns, with looks to the target pictures starting to increase rather steeply from about 600 ms onwards in both the direct and the indirect evidential conditions. The monolingual group’s proportion of looks to the target picture reached a peak at around 1200 ms. After 1200 ms, the monolinguals started turning their gaze to the context picture, where the actions were shown to be in progress, in the direct evidential condition. They kept fixating the target photo during the processing of indirect evidentials in the same time window. Therefore, on the basis of this visual inspection, two time windows were chosen for the statistical analyses: (i) the ‘main’ time window (200–2000 ms), and (ii) a ‘late’ time window (1200–2000 ms); see **Figure 3**.

The fixed effects of the mixed-effects logistic regression models built on the proportion of looks data from the main and late time windows are shown in **Table 4**. Since proportion of looks data do not display a linear relationship with time, in addition to linear time, quadratic, and cubic time variables were included in the models so that fixation changes over time can be best captured.

**Table 4:** Fixed effects from the mixed-effect logistic regression model performed on the proportion of looks data in the main time window (200–2000 ms) and late time window (1200–2000 ms).

	Main time window (200–2000 ms)				Late time window (1200–2000 ms)			
Fixed effect	Estimate	*SE*	*t*-value	*p*-value	Estimate	*SE*	*t*-value	*p*-value
Intercept	5.674	1.092	5.198	<0.001***	-4.241	8.824	-0.481	0.631
Linear time	-1.012	1.088	-0.001	0.999	2.123	7.795	0.027	0.978
Quadratic time	2.451	2.800	8.753	<0.001***	-1.320	1.053	-1.253	0.210
Cubic time	-9.140	9.202	-9.933	<0.001***	2.648	2.193	1.207	0.227
Condition (indirect evidential)	-4.365	4.764	-0.916	0.360	-2.148	7.260	-2.958	0.003**
Group (early bilingual)	-7.072	2.459	-2.876	0.004**	-1.252	3.081	-4.063	<0.001***
Group (late bilingual)	-5.331	2.426	-2.197	0.028*	-1.023	3.041	-3.364	0.001**
Condition × Group (early bilingual)	5.432	6.944	7.823	<0.001***	1.018	1.060	9.601	<0.001***
Condition × Group (late bilingual)	3.246	6.883	4.716	<0.001***	5.260	1.051	5.003	<0.001***
	Formula: PropLook ∼ Linear time + Quadratic time + Cubic time + Condition * Group + (1 + Linear time | participants) + (1 + Linear time | items)					

*p < 0.05, **p < 0.01, ***p < 0.001.

Outcomes from the model for the main time window showed significant effects of group, with both early and late bilinguals fixating less frequently on the target picture within the main time window compared to the monolinguals. Significant interactions between condition and group were found which indicate between-group differences in participants’ eye-movement patterns across the two experimental conditions.

Within the main time window, fixations on the target picture were found to be reduced in the direct evidential condition in both the early (β = 0.0518, *SE* = 0.0052, *z* = 9.857, *p* < 0.0001) and late bilinguals (β = 0.0253, *SE* = 0.0051, *z* = 4.911, *p* < 0.0001) in comparison to the number of target fixations in the indirect evidential condition. The monolingual group showed no difference between the two evidential conditions (β = -0.0046, *SE* = 0.005, *z* = -0.919, *p* = 0.35), as was confirmed by Tukey tests.

The early bilinguals fixated less on the target picture than the monolinguals in the direct evidential condition (β = -0.09448, *SE* = 0.03616, *z* = -2.613, *p* = 0.024), while the late bilinguals differed only marginally from the monolinguals here (β = -0.07704, *SE* = 0.03554, *z* = -2.168, *p* = 0.077). The late and early bilinguals did not differ from each other in the direct evidential condition (β = 0.01744, *SE* = 0.03617, *z* = 0.482, *p* = 0.87), however. For the indirect evidential condition, no between-group differences were found (all *p*s > 0.67).

For the late time window (see **Table 4**), the model outputs showed effects of condition, group, as well as interactions between these two factors. To investigate the nature of these differences, *post hoc* analyses were performed. During their processing of direct evidentials, both late (β = -0.11413, *SE* = 0.04053, *z* = -2.816, *p* = 0.013) and early bilinguals (β = -0.12507, *SE* = 0.04115, *z* = -3.040, *p* = 0.006) looked less frequently toward the target picture than the monolinguals did. Again, no significant between group differences were found during participants’ processing the indirect evidentials (all *p*s > 0.44).

Within-group comparisons revealed that both the early (β = 0.077061, *SE* = 0.0077, *z* = 9.98, *p* < 0.0001) and the late bilinguals (β = 0.034811, *SE* = 0.0075, *z* = 4.599, *p* < 0.0001) fixated more frequently on the target picture in the indirect than in the direct evidential condition during the late time window. The monolinguals showed the opposite pattern: they looked at the target picture slightly more frequently in the direct than the indirect condition (β = -0.015209, *SE* = 0.0072, *z* = -2.017, *p* = 0.035).

Notwithstanding the monolingual participants’ overall higher number of fixations on the target picture in the direct evidential condition in the late time window, they tended to shift their gaze toward the context photo from about 1200 ms in the direct evidential condition whereas they kept fixating on the target photo in the indirect evidential condition (see **Figure 3**). To further examine these eye-movement changes over time, we ran the model again on the monolingual eye-movement data from the late time window with fixed effects of linear time and condition. The model output showed a significant effect of linear time (β = -1.243, *SE* = 2.094, *t* = -5.937, *p* < 0.001), condition (β = -1.954, *SE* = 4.80, *t* = -4.071, *p* < 0.001), and an interaction between the two factors (β = 1.128, *SE* = 2.966, *t* = 3.804, *p* < 0.001). These results confirm that the monolinguals’ fixation changes over time within the late time window were different in the direct and indirect evidential conditions.^5^

### Summary of Results

Both the late and the early bilinguals were slower and less accurate than the monolinguals in their responses in the direct evidential condition, whereas they patterned with the monolinguals in the indirect evidential condition. Furthermore, within the response data there were interactions with group, showing that both the late and early bilinguals responded less accurately to the direct than to the indirect evidential condition, while the monolinguals showed no difference between these two conditions. A similar contrast was found in response latencies.

These behavioral results were reflected in the proportion of looks data. Bilinguals were less likely to look at the target picture in the direct compared to the indirect evidential condition in both the main and the late time windows. In the late time window (i.e., from 1200 ms onwards), the monolinguals shifted their gaze toward the context picture during their processing of direct evidentials, whilst the bilinguals’ eye-movements tended to oscillate more between the target and context photos.

## Discussion

The results reported add to our understanding of how evidential morphology is processed and linked to the type of evidence available by both mono- and bilingual Turkish speakers. Our first research question was whether bilinguals differ from Turkish monolinguals in processing evidentiality. The second question was whether monolingual, late and/or early bilingual Turkish speakers differ in their processing of direct vs. indirect evidentials.

The answer to the first question is clearly positive, as early and late bilinguals were found to differ from the monolinguals in their end-of-trial responses and eye-movement patterns. Both late and early bilinguals responded less accurately and looked less often to the target picture when processing direct evidentials compared to the monolinguals. Regarding our second research question, we observed an interesting asymmetry between the direct and indirect evidential conditions in the two bilingual groups that was absent in the monolingual group. Both early and late bilinguals showed greater problems processing direct compared to indirect evidentiality. This asymmetry was reflected in reduced response accuracy, longer response latencies, and in a lower proportion of looks to the target picture, in the direct compared to the indirect evidential condition. No statistical between-group differences were found for early vs. late bilinguals, indicating that the onset of bilingualism did not affect the way they processed evidentiality.

How can the observed pattern of results be accounted for? Previous studies have shown that bilinguality may affect the way people use or process their native language, with bilinguals – in particular, heritage speakers – often performing differently from monolinguals on linguistic tasks. The age of bilingualism onset has been argued to be an important factor: whilst non-target like performance in late bilinguals is often attributed to first language attrition, non-target like performance in early bilinguals has been associated with incomplete acquisition. In first language attrition, individuals who initially acquired their native language fully may lose certain properties of that language later in life, possibly influenced by properties of a second language. In incomplete acquisition, by contrast, early bilinguals (or heritage speaker) experience disrupted acquisition processes, as a result of which certain properties of their native language are never properly acquired.

In Turkish child language acquisition, the indirect evidential is acquired after the direct evidential; it is conceivable that our early bilinguals did not fully acquire the correct use of indirect evidentials as compared to the late bilinguals. Incomplete acquisition in early bilinguals has also been associated with more severe outcomes in comparison to attrition in late bilinguals (Montrul, 2002, 2008). This is not what we found, however. Both bilingual groups were at the monolingual level in processing indirect evidentiality but performed worse than the monolinguals in the direct evidential condition. We did not find any differences between early and late bilinguals’ responses in the direct evidential condition, which means that both bilingual groups were equally affected in their processing of direct evidentiality in comparison to the monolinguals. Our results, thus, do not indicate that an earlier onset to bilingualism results in more severe effects than a later onset of bilingualism.

We believe that the late bilinguals in our study were affected by a form of attrition. However, on the basis of the current data, for the early bilinguals it is impossible to precisely tease apart effects of attrition from those of incomplete acquisition. Studies on monolingual children’s acquisition of evidential morphology are still scarce. These studies suggest that by the age of six, the conceptual development linked to the use of indirect evidential forms is not yet fully complete (e.g., Öztürk and Papafragou, 2007, 2008). It is thus unclear at which age the development of the evidential system finalizes. The fact that both bilingual groups showed reduced sensitivity to direct evidentials but were at the monolingual level in their processing of indirect evidentials indicates that the representation and/or pragmatic function of the direct evidential morpheme differs between mono- and bilingual Turkish speakers. This suggests that the underlying reason for the observed between-group differences is not related to the age at which the bilinguals’ acquired German but to the linguistic properties of evidentiality.

Recall that Turkish indirect evidentials are assumed to have modal properties unlike direct evidentials, and that the former are thought to be semantically more complex that the latter. Turkish linguists also agree that the direct evidential is the ‘unmarked’ evidential form (e.g., Aksu-Koç, 1988, 2000; Sezer, 2001; Johanson, 2006), while the indirect evidential is the more marked term in its semantics. Given Montrul’s (2009) finding of Mood distinctions being more strongly eroded than non-modal inflectional distinctions in Spanish heritage speakers, we expected bilinguals’ sensitivity to indirect evidential markers to be more reduced than their sensitivity to direct evidential markers. Difficulty with indirect evidentials is also what the Interface Hypothesis predicts. According to this hypothesis, bilinguals tend to have problems with integrating information from multiple linguistic levels at the syntax-discourse interface and thus should show more difficulty processing marked compared to unmarked forms (e.g., Sorace and Serratrice, 2009). However, both early and late bilinguals were more accurate and quicker to respond to the more marked term (the indirect evidential) here, whose use is licensed only by the availability of a specific type of evidence, than to the less marked term (the direct evidential) in the current study.

Alternatively, we may be able to account for our findings by assuming that, even though Turkish heritage speakers are aware of the semantic and pragmatic properties of indirect evidentials, the direct evidential morpheme -DI has become the default form for referring to past events regardless of information source. That is to say that the bilingual participants take the direct evidential to be a past tense marker without any specific evidential content, whilst they retained the indirect evidential as an evidential form associated with reporting non-witnessed events. This hypothesis broadly fits with Arslan et al. (submitted) finding that early bilingual speakers of Turkish were largely insensitive to mismatches between evidential verb forms and evidential contexts but had retained sensitivity to incorrect tense forms. Although the early bilinguals examined by Arslan et al. (submitted) seemed unable to identify information source violations for either of the two evidential forms, Arslan and Bastiaanse (2014) found an asymmetrical substitution error pattern. The early bilingual speakers of Turkish mistakenly produced direct evidential forms in contexts where an indirect evidential would normally be required. This indicates that the early bilinguals ignored the evidential content of direct evidential forms, using these forms to refer to the past irrespective of whether or not its use was licensed by the type of evidence available. This is also supported by the current findings. When given a visual depiction of directly witnessed evidence for an event, bilingual speakers of Turkish have more problems processing direct evidential forms than monolinguals, whereas they are no different from monolinguals in their processing of indirect evidentials accompanied by a visual depiction of indirect (inferential) evidence.

Recall that one idea behind the conceptual design of this study was to reveal whether and when speakers of an evidential language consider the evidence during processing grammatical evidentiality. That is, we were also interested in whether the speakers were aware of the evidential implications signaled by the verbal forms. Both the behavioral and eye-movements data point in the same direction: both late and early bilinguals fixated less frequently on the target picture in the direct than in the indirect evidential condition, whereas the monolinguals showed no difference between these two conditions in the main time window. Fewer looks to the target picture in the direct evidential condition means that the bilingual participants fixated more often on the context picture in the direct than in the indirect evidential condition in both the main and late time windows. They also clicked on the context picture more frequently in the direct evidential condition, as shown by their reduced response accuracy. This was not what the monolinguals did. In the late time window, although the monolinguals tended to look at the target picture slightly more often in the direct evidential than the indirect evidential condition, they were equally able to choose the target picture in both conditions. This indicates that the bilinguals were less likely to recognize that the context pictures merely provided a form of evidence, and more likely to mistake the context picture for the target picture, in comparison to the monolinguals.

The time course of participants’ eye-movements during processing direct evidentials also differed between the monolingual and bilingual Turkish speakers. The monolinguals shifted their gaze toward the context picture, where the action was shown to be in progress, in the late time window (from about 1200 ms) while processing direct evidentials. This suggests that increased looks toward the context picture allowed the monolinguals to verify that the action could indeed be ‘witnessed’ directly, compatible with the use of a direct evidential form. This shift was less prominent in the two bilingual groups, although their fixations also changed over time in the late time window due to larger oscillations between the two pictures (see **Figure 3**), indicating that the bilinguals felt less of a need to ‘witness’ the action, and thus, to verify whether the use of a direct evidential was warranted. This suggests that the direct evidential has been subject to semantic or pragmatic ‘bleaching’ in Turkish heritage grammars, making it appropriate for use in both ‘witnessed’ and ‘non-witnessed’ types of evidential contexts. Examples of a restructuring of grammatical systems in bilingual speakers of minority languages (i.e., heritage speakers) are not in fact uncommon. Polinsky (2006), for instance, reports simplifications in the gender and aspect systems of Russian heritage speakers, and Kim et al. (2009) observed a simplification of the pronominal system in Korean heritage speakers. However, whether or not the apparent erosion of evidentiality distinctions in Turkish heritage speakers is triggered by prolonged exposure to the majority language of our bilingual participants cannot be determined in the absence of a bilingual comparison group whose L2 is typologically different from German (and Dutch).

## Conclusion

Our results show that both early and late Turkish/German bilinguals differed from Turkish monolinguals in their processing of direct (but not indirect) evidentiality. These data do not support the Regression Hypothesis or the Interface Hypothesis. We have argued that our findings can be accounted for by assuming that the bilinguals take the direct evidential to be the ‘unmarked’ default form for referring to past events, in line with what has previously been reported by Arslan and Bastiaanse (2014) and Arslan et al. (submitted). Taken together, our findings from the production, off-line comprehension and online processing of evidentiality by Turkish-German and Turkish-Dutch bilinguals provide converging evidence suggesting that the grammar of evidentiality in these bilinguals has simplified at the representational level. The bilinguals under study are, however, aware that the use of indirect evidential forms is linked to a particular type of evidence, as both our behavioral and eye-movement data suggest that the early and late bilinguals interact with the indirect evidence in a similar way as the monolinguals.

## Author Contributions

Conception or design of the experiment: SA, RB, CF; acquisition and analysis of the data: SA; drafting of the manuscript: SA; revising of the manuscript: RB, CF; final approval of the content: SA, RB, CF; agreement to accuracy or integrity of any part of the work: SA, RB, CF.

## Conflict of Interest Statement

The authors declare that the research was conducted in the absence of any commercial or financial relationships that could be construed as a potential conflict of interest.

## Footnotes

1 A reviewer suggests that the use of evidential sentences with the padding phrases positioned at the end of the sentences sounds rather unnatural, especially for the indirect evidential sentences. The reviewer claims that the indirect evidentiality sentences used in the current study cannot be combined with adverbial phrases such as *ender bir istekele* ‘with unusual desire’ since the indirect evidential signals a “non-witnessed” event. This is on the assumption that in inference contexts, where there is nobody who actually witnessed how the action was performed, adverbials of this kind cannot be used to modify the action. The purpose of our oﬄine rating task was to ascertain whether our direct and indirect evidentiality stimuli sounded equally plausible.

2 The mean onset of the critical verbs was 4162 ms after each trial began, minus 1000 ms silence, and the mean sentence offset time was as 5470 ms from the beginning of the sentences.

3 An initial model was built with future tense items included, which showed no effects of condition for indirect evidential vs. future tense items (β = -0.501, *SE* = 0.289, *z* = -1.731, *p* = 0.082), and for direct evidential vs. future tense (β = -0.528, *SE* = 0.286, *z* = -1.840, *p* = 0.065). Effects of group were not found, as well: late bilinguals vs. monolinguals (β = -0.3109, *SE* = 0.3758, *z* = -0.827, *p* = 0.40), and for early bilinguals vs. monolinguals (β = -0.4961, *SE* = 0.3752, *z* = -1.322, *p* = 0.18). As the future items were used as distractors, they were omitted from all subsequent analyses.

4 The accuracy of responses in the direct and indirect evidential conditions in the late bilingual group correlated with their Turkish (*r* = 0.102, *p* = 0.041) and German (*r* = 0.184, *p* < 0.001) language proficiency scores, whereas no such correlations were found in the early bilingual group (both *p*s > 0.36), as shown by Pearson tests.

5 Participants’ eye-movement changes over time in the late time window also showed different group characteristics within each condition. In the direct evidential condition, there were effects of linear time (β = -1.053, *SE* = 1.287, *t* = -8.181, *p* < 0.001), and of group (β = -6.760, *SE* = 2.542, *t* = -2.659, *p* < 0.01). In the indirect evidential condition, by contrast, there was an effect of linear time (β = 3.844, *SE* = 1.259, *t* = 3.054, *p* < 0.001) but not of group (β = -2.731, *SE* = 13.317, *t* = -0.823, *p* = 0.41). Eye-movements changed over time in both condition, as linear time was significant in both conditions. However, there was an effect of group in the direct evidential (but not in the indirect evidential) condition suggesting that the moment-by-moment eye-movements changes in the late time-window are different for individual groups in the direct evidential condition, but similar in the indirect evidential condition.
